# Recognition rights, mental health consumers and reconstructive cultural semantics

**DOI:** 10.1186/1747-5341-7-6

**Published:** 2012-01-13

**Authors:** Jennifer H Radden

**Affiliations:** 1 Philosophy Department, University of Massachusetts Boston, Morrissey Boulevard, Boston, Massachusetts 02125, USA

## Abstract

**Introduction:**

Those in mental health-related consumer movements have made clear their demands for humane treatment and basic civil rights, an end to stigma and discrimination, and a chance to participate in their own recovery. But theorizing about the politics of recognition, 'recognition rights' and epistemic justice, suggests that they also have a stake in the broad cultural meanings associated with conceptions of mental health and illness.

**Results:**

First person accounts of psychiatric diagnosis and mental health care (shown here to represent 'counter stories' to the powerful 'master narrative' of biomedical psychiatry), offer indications about how experiences of mental disorder might be reframed and redefined as part of efforts to acknowledge and honor recognition rights and epistemic justice. However, the task of cultural semantics is one for the entire culture, not merely consumers. These new meanings must be *negotiated*. When they are not the result of negotiation, group-wrought definitions risk imposing a revision no less constraining than the mis-recognizing one it aims to replace. Contested realities make this a challenging task when it comes to cultural meanings about mental disorder. Examples from mental illness memoirs about two contested realities related to psychosis are examined here: the meaninglessness of symptoms, and the role of insight into illness. They show the magnitude of the challenge involved - for consumers, practitioners, and the general public - in the reconstruction of these new meanings and realities.

**Conclusion:**

To honor recognition rights and epistemic justice acknowledgement must be made of the heterogeneity of the effects of, and of responses to, psychiatric diagnosis and care, and the extent of the challenge of the reconstructive cultural semantics involved.

## Introduction

Those in mental health-related consumer movements have made many of their demands clear, including the demand for humane treatment and basic civil rights, an end to stigma and discrimination, and a chance to participate in their own recovery. But theorizing about identity politics and the politics of recognition suggests that they also have a stake in something else: the way they and their conditions are to be depicted in the broadest terms. In the following, I want to sketch some of the real world imperatives that are implied in this suggestion, employing the work of political philosopher Nancy Fraser and appealing to first person narratives about the experience of mental disorder as source material. This is not primarily a theoretical discussion, but rather one that attempts to apply theory to a pressing social and political phenomenon. The first part of this discussion explains why some demands of those who receive, or have received, mental health care can be seen as demands for recognition rights, and describes the task of reconstructive cultural semantics apparently called if we honor the recognition rights of mental health care consumers. Some challenges implicit in that task are introduced (see Results). First person accounts of mental disorder expose conceptions of insight and meaning at odds with the precepts and assumptions to be found in the 'master narrative' of medical psychiatry. In light of these contested realities, it is argued that the reconstructive task will involve negotiation over the contested meanings involved.

### Mental health consumers' movements

The broad details about mental health consumers' movements are familiar to us. Modeled on other consumer and liberation movements, these political interest communities are variously self-identified as "service users," "survivors," "consumers," "mad pride," and "recovery" groups. Their efforts to achieve basic civil rights, noted above, have taken a range of forms and met with uneven, but promising, success. (In what follows, the term "mental health care consumers" refers to members of these groups, whose commonality is receiving or having received mental health care.) One aspect of those movements is emphasized in this discussion: they have fostered, and drawn attention to, an old, but today burgeoning, literary genre - published memoirs as well as less formal descriptions recounting the experience of mental disorder from the perspective of the first person. These accounts, I will argue, represent an important starting point in the quest for recognition; moreover, they help us understand some of the challenges involved in a proper recognition of those who have received psychiatric diagnoses.

### Redistributive and recognition paradigms

Important theorizing around what is known as identity and recognition politics has come from political philosophy in recent decades. (See, for example, [1-3].) Influenced by German critical theorists working in a Hegelian tradition, such as Habermas, Nancy Fraser identifies two distinct and irreducible paradigms of justice [4]. The first is the familiar redistributive paradigm associated with economic justice. As well though, Fraser identifies a "recognition" paradigm. This latter paradigm requires the elimination of *cultural injustices rooted in cultural patterns of representation, communication and interpretation*. To expunge culturally constructed identities that the people to whom they are attributed want to reject, Fraser advocates that "some sort of cultural or symbolic" change must take place. In additional to the social and economic change dealt with in the redistributive paradigm, this cultural or symbolic change will be seen as a remedy for cultural, as distinct from merely economic, injustice. (See [4] page 15.)

The particular groups which Fraser uses as her examples in this discussion are those based on class, gender, race, and "despised sexualities." But with its emphasis on a liberty right to group identity recognition, Fraser's appeal to a recognition paradigm can be seen as an expression of identity politics, which is far from limited to those particular categories (class, gender, race, despised sexualities). It is a template for any of the groups participating in the identity politics of today (battered women, the adult children of incest perpetrators, and those with physical disabilities, for example). In addition, and my particular concern here, it may have application to the consumers of mental health services.

Recognition is a normative status, demanded by justice, and sensitive to variations among those for whom it is sought. Remedies for disadvantaged and marginalized groups will reflect the differences between such groups, including the respective injustices to which they have been subject. Thus the systematic misrepresentation suffered by people with psychiatric diagnoses will require a more thoroughgoing and fundamental remedy than will the lack of recognition suffered by some other marginalized groups. In fact, it is not a failure of recognition as such, that the mentally ill have been prey to, it is almost the reverse - they have been all too conspicuous. What they have suffered has been a notably systematic and damaging *mis-recognition*. Mental disorder has been allied with otherness, with irrationality, lack of competence, deficient agency, identity and even humanity. Its sufferers have also been the victims of what has been described as epistemic injustice - deprived of semantic authority and credibility (See [5]).

Fraser is sparing in her account of what forms the cultural and symbolic change that she advocates might take, and we must extrapolate to the particular case of the group identity of the consumers of mental health care. In Fraser's analysis, acknowledgement of the recognition paradigm will proceed in two stages. A remedy for the cultural injustice, or mis-recognition, visited upon some group would involve, in her terms, a self-generated, symbolic redefinition of their group identity. And the second stage: this redefinition would be so powerful as to eventually eliminate generalities about such group identity and allegiances. The goal of the recognition paradigm remedy would be to "put the group out of business as a group," in her words (See [4] page 19).

The applicability of the first of these steps to mental health consumers is the one I want to consider here. Assimilationist assumptions of the kind on which the second goal rests have been challenged even in relation to the groups based on class, gender, race and despised sexualities Fraser names. And as they have identified themselves thus far, mental health consumers do not speak with one voice over whether their ultimate goals are to eliminate and de-emphasize their commonalities until assimilation is achieved, or to emphasize and valorize them.

Fraser rightly places the two paradigms, the one economic and the other cultural and symbolic, on an equal footing. And injustices associated with the first, economic paradigm will be equally applicable to the consumers of mental health care. Economic deprivations resulting from stigma over mental illness - widely acknowledged - include access to mental health care, and housing, employment and other forms of discrimination with economic implications. Undeniably, the redistributive paradigm calls for equal attention and acknowledgement, but because it has already been at the forefront of these movements' demands, that paradigm can also be set aside here.

In more recent writing, Fraser has noted some of the dangers of equating the politics of recognition with identity politics, and we'll turn to those concerns at the end of this discussion. But it is with the group identity of mental health care consumers in relation to recognition that I want to begin.

### The right to recognition for mental health care consumers

If we think about the collective identity of consumers of mental health care in light of Fraser's recognition paradigm, several things emerge. (1) If there is a right to recognition of this kind, then as part of such a right those making up these movements have an interest in the manner in which their experiences and lives are represented. Due to the degree of stigma and discrimination associated with cultural patterns of representation, communication and interpretation about mental disorder in particular, that interest seems likely to be a compelling one, and quite as important to consumers as their interest in achieving the more usual liberty rights they have demanded. Moreover (2) this must be, as Fraser puts it, a self generated effort. It is one that, in the spirit of the consumer movement slogan "Nothing about us without us," and the model of other liberation movements, calls for group member *participation *[6]. Consumers must themselves take part in the symbolic redefinition of their group identity.

Efforts at symbolic redefinition associated with identity politics take diverse forms. In the simplest cases, redefinition involves name change (from American Indian to Native American, from Disabled to Differently Abled, from Victim to Survivor, for example). The shift from "Mentally Ill" to "Survivor" and "Recovering" has to some extent been the kind of group-generated response Fraser envisions. There cannot be found a unified purpose in these all these name changes, however, as the shift proposed by some from "Mentally Ill" to "Mad Pride", makes particularly clear. The differing connotations of "survivor" (with its implicit critique of the care received); "consumer" and "service user" (stressing commonalities with other recipients of goods and services); and "mad pride," with its impulse to rehabilitate and valorize, reflect profoundly different attitudes. But whatever it conveys, a mere name change - even if agreed upon, and even if necessary - must prove insufficient here, in light of the historically entrenched and pervasive stigma and discrimination associated with cultural patterns of representation, communication and interpretation about mental disorder, and the previous voicelessness of its sufferers in public discourse.

So the larger challenge calls for re-conceptualizing all aspects of the way mental disorder is construed, not merely the terms used to refer to it. Moreover, this challenge demands redefinition that goes beyond the merely personal. Not only individuals' autobiographical stories, their symptoms, their conception of their own disorder and the treatment received in relation their own lives, although these are all important, but broader conceptions of mental health, illness, rationality, responsibility and competence must all be subject to re-envisioning in this effort. The additional political goal involves what might be called *reconstructive cultural semantics*: a revision which consumers undertake - if not alone, then as central, and privileged, participants. - of these most general ideas about sanity and madness.

The scope and challenge of this cultural project can hardly be overestimated. First, much is implicated in a reconstruction of cultural ideas about mental health and illness, because the beliefs, metaphors, assumptions, and presuppositions affecting patterns of representation, communication, and interpretation about this kind of disorder are entwined with categories and concepts fundamental to our cultural norms and values: rationality, mind and character, self-control, competence, responsibility and personhood. The position of epistemic disadvantage from which the central participants in this project (the consumers) must proceed, is an additional aspect of the challenge it presents. The mad have been excluded from the epistemic as well as the social community, their voices disregarded and dismissed as meaningless. Their struggle must include being believed as credible knowers, as well as merely being heard. And finally, as we shall see, there are seemingly incompatible perspectives and contested realities involved. Being heard at all, and being accorded the status of credible, are the demands made by many marginalized groups. But in addition to surmounting these obstacles, consumers of mental health care will need to negotiate the controversial ideas and contrary perspectives they bring to more mainstream and widely accepted understandings.

## Results

### Reconstructive cultural semantics: action steps

First person accounts of experience with mental illness, mental health care and psychiatric diagnosis provide a valuable source material, and a remarkable and apposite guide in the effort to begin this broader project of redefinition. Such "madness narratives" range from full memoirs to briefer first person accounts and comments, and they have in recent years come to be recognized as constituting a distinctive literary genre, deserving of critical attention. In English alone even published works have been assessed to number in the hundreds, and countless others, it has been observed, "lie half-written in desk drawers, or unacknowledged in physicians' publications and case notes." ([7] See page 01). There have always been such memoirs, almost since we have autobiographical records of any kind. The *Booke of Margery Kemp*, for example, dictated by an illiterate woman subject to recurrent episodes of distressing disorder, dates to late medieval times. Today has brought a burgeoning increase in such documents, as well as some critical literary attention (See [8,9]). Only consumers themselves can provide testimony and bear witness *in this way*. However sympathetic and knowledgeable they might be, non-consumers are differently positioned, with obligations lying elsewhere. As allies, non-consumers must acknowledge, honor and attend to these first person narratives of survival, recovery, and experience with disorder, psychiatric diagnosis, and mental health care, and remain open to the potential cultural transformation they can bring; they must confer credibility on consumers as knowledge claimants. In addition, as we shall see presently, non-consumers and particularly mental health caregivers may need to participate in a negotiation over the contested realities that are revealed by some of this writing.

First person accounts of mental disorder have often taken the form of resistance writing, it has been observed, once created despite and in opposition to the powerful forces of the asylum in which their authors have been involuntary and reluctant inmates, and of the societal and medical systems that have placed them there. (They have for this reason been analogized to slave narratives [7].) Even in today's era of deinstitutionalization and more effective treatments moreover, these memoirs remain highly transgressive documents, with wide-ranging political implications. One focus of the more contemporary memoirs introduced here is their relation to the medical "master narrative" within which they are regularly - yet not entirely or unambiguously - framed. This is a critical aspect of mental health memoirs because of the power of the medical psychiatric structures and institutions within which diagnosis and treatment usually occur today. These documents hold other interest and value as well. However, their relation to medical framing exemplifies the depth and pervasiveness of attitudes at the heart of the re-framing and re-definition challenges involved in any task of reconstructive cultural semantics, and they will be our focus here. First person accounts of the experience of mental disorder are invaluable in helping us understand its effects on the personal lives of its sufferers. But its medical framing, in which mental disorder is depicted as an objective biological and behavioral phenomenon, provides a more general, public, and purportedly objective analysis, and a thoroughgoing reconstruction must address cultural meanings at this level of generality, publicity, and purported objectivity.

### The medical narrative as "master narrative"

Memoirs from our era regularly introduce reductionistic assumptions from modern biological psychiatry. Mental aberration is pathology, or dysfunction, and psychiatric symptoms are the meaningless causal products of a disordered brain, best expunged by medical science. Such medical framing demands attention for two reasons, its prevalence, and its compelling narrative force.

The same sort of framing that is found in memoirs of psychiatric diagnosis and disorder governs accounts of bodily illness and disability, of course. And in order to grasp the ubiquity and power of the medical "master narrative," we can begin with first person accounts of more ordinary disease and disability. Analyzing his personal experience with heart disease and cancer, Arthur Frank writes of the way medical discourse dictates the terms and reality in which all illness and disability memoirs are cast. A cost of the "sick role," he asserts, is nothing less than a "narrative surrender" [10]. Telling it medically, Frank suggests, is taken as telling it "how it is." Medical language and presuppositions function to rob alternative framings of rhetorical vitality and even intelligibility.

Conditions like heart disease and cancer for the most part leave the patient still possessed of mental faculties and the communicative and other capabilities we associate with personhood, and yet this curb on narrative vitality and intelligibility is experienced and is, if Frank is right, next to unavoidable. So much more should we expect the sort of narrative "surrender" he describes when the patient's very disorder is understood to jeopardize capabilities at the center of conceptions of personhood and justify a failure to confer credibility to its sufferers. And medical framing, we shall see, pervades psychiatric memoirs through and through.

Attitudes towards medical framing are another feature shared by psychiatric memoirs and the narratives that describe bodily disease and disability. Authors of both kinds of writing chafe at, and attempt to resist, that framing, as Frank did. This resistance has been depicted as a struggle against what those discussing disability writing have sometimes called "master narratives," that is, "repositories of common norms" which "exercise ... authority over our moral imaginations"([11] page 8). Master narratives are an expression of the power of a dominant group; it is a power so great as to create conditions for non-recognition or mis-recognition of marginalized groups such as, in our case, those bearing psychiatric diagnoses. And those who identify master narratives offer a prescription: illness and disability memoirs ought to provide "counter -stories" to unseat the prevailing master narratives and weaken their power. Counter-stories have been described as those that resist an oppressive identity and attempt to replace it with one that commands respect; they challenge unjust assumptions implicit in the master narrative and identify their subjects "more accurately and fairly" as the result (See [11] page 6.)

If we look at the history of medicine, we can see that medical framing about mental disorder itself once constituted a counter-story. At the beginning of the modern era, it would have been told in defiance of a master narrative depicting such disorder as divine punishment, or demonic possession. An example of this is John Perceval's 1840 *Narrative on the Treatment Experienced by a Gentleman during a State of Mental Derangement *[12]. Likening the mind to "an excellent piece of machinery" he repudiates the religious and moralistic interpretations of his disorder that were the orthodoxy of his day. In today's writing, however, the medical framing represents a master narrative. Psychiatric memoirs - due both to the fact that they are there at all, and to the way they are able to acknowledge yet resist and challenge medical discourse, can be seen as "counter-stories."

### Reading between the lines: counter stories about insight and meaning

Some acknowledgement of the medical understanding with which their care givers approach their condition seems almost inescapable in these memoirs. Imaginative flexibility and rhetorical impact must be affected because of the ubiquity, authority, and influence of the master narrative of medical psychiatry. The vitality and coherence of psychiatric memoirs cannot but be constrained by these powerful psychiatric paradigms. They deflate the very purpose of such writing. If ideas are delusional, perceptions are mistaken, feelings are deemed unmoored, imaginary, or inappropriate, behavioral responses are the meaningless output of a disordered brain, what value can such first person accounts be accorded? When written in the throes of active episodes of disorder, they are further symptoms, or harmless ways to fill the time until reason returns - occupational therapy on the cheap. When constructed after the fact, they are understood as meaningless irruptions in the flow of a comprehensible life "narrative," better forgotten. Within the master narrative, psychotic episodes are at most opportunity costs in a more functional life trajectory, and are no more meaningful than a bad dream. This includes, but goes beyond the usual epistemic injustices inflicted on members of marginalized groups by the broader society. Moreover, it reflects sources of power that are augmented through institutionalized expertise and authority.

That said, *total *narrative surrender such as Arthur Frank speaks of, is rare in these memoirs. Occasionally, they also fail to find meaning or value in the experiences and symptoms they record. But this is unusual. Much more commonly, while constrained by medical psychiatric framing, these narratives reveal their authors' chafing at, and challenging, its limitations. Often however, we must look between the lines to find the counter- stories.

The ways today's memoirists acknowledge the influence of these biomedical presuppositions are various. They are told in a range of tones, for example - the indignant, the resigned, the sardonic and satirical; they are in varying degrees accepted, or railed against; they are deemed to be incomplete explanations - and also explanatorily irrelevant, and beside the point. Rarely, in all the variation is it accepted that medical presuppositions offer the only accurate, exhaustive and useful perspective from which to understand and explain the experience of disorder, however. Instead, we find observations like the following, written by a woman treated for schizophrenia. "Scientific drug therapy and a life of faith" she explains, "...have worked together in my recovery...Along with taking this medication I have sought help from my religion...It is a combination of resperidone and religious practice that gives me clear thinking" ([13] page 543.) Room is made in this account for the effects of biological psychiatry, but the author emphasizes that the resperidone was only one of several factors responsible for her improvement. In another passage, the same author attributes meaning and significance to her psychotic symptoms. "Once during a storm, the rain was falling in a heavy downpour, and the sound of it falling became a voice. It said "Believe in Jesus Christ and you will be saved. This message within the rainfall and the call to faith it invoked have been important factors in my recovery. A dramatic improvement in my mental health has resulted from this message. In general, the psychiatric community has been reluctant to acknowledge anything of value in psychotic phenomena, but I know in an experiential way that meaning can sometimes be found in them" (See [13] page 546.)

The notion that "reluctance to acknowledge anything of value in psychotic phenomena" is *belied by the patient's own experiences*, finds its way in these accounts again and again. Here is another: "I don't believe that the voices and hallucinations from which I've suffered in my life have merely been the result of a 'biochemical imbalance,' but have reflected a need from deep within myself to find myself" ([14] page 148.) Rather than the meaningless byproducts of a disordered brain, psychiatric symptoms are here depicted as meaningful elements of experience and identity.

In addition, patient accounts often reveal "meaning making" that, while idiosyncratic, is nonetheless of the utmost personal significance to the patients themselves, their conceptions of their lives, and even, perhaps, the course of their condition. Describing her experience of a brain scan one woman, who had been hospitalized for many years with psychosis, spoke of her (seemingly unlikely) change in appearance since the scan, and other changes as well:

Yes I have [changed]. My soul is red now. It used to be black. Everything is easier now. I can breathe. I can't explain it, but I feel happy. I'm satisfied with myself...I'm burning with a love for life. I accept life now."([15] page 198.)

Arguably, the "meaning" this woman finds in her experience is so idiosyncratic, and lacking in intersubjective coherence as to be, in one sense, meaningless (despite its proper grammar). Yet whatever its lack of "objective" or intersubjective meaning, or its relative importance, her experience of the brain scan possesses associations and connotations of strong significance to her. In this respect, at least, it cannot be dismissed as the undesirable, dispensable state, ideally expunged, that seems to be presupposed by much contemporary medical understanding of such symptoms. Meaningfulness and even intelligibility, this writing seems to indicate, rest on more than mere inter-subjective agreement. And even an idiosyncratic meaning system - what has sometimes been dismissed as a merely private "language" and thus no language at all - can still have intra-personal effects. Too little is yet understood about healing in psychiatry for us to dismiss out of hand that this patient's ideas might serve to ameliorate her condition. (Reports from the Hearing Voices Network seem to provide some confirmation of this general claim. Surveys showed that strategies of actively engaging with inner voices - by asking what lessons these messages brought, for example, served their hearers better than ignoring them [16].) Like narratives that emphasize there are different explanations of psychotic symptoms, this passage suggests what recent findings in anthropology confirm: that such symptoms may be meaningful in more than one way [17,18].

To the rhetorical force of the master story that serves to rob any counter-stories of narrative vitality, must be added another reason why we often have to read between the lines here. Clinical lore designates lack of medical insight an indication of illness.

Sometimes absence of insight is presented as itself a *symptom *of the severe disorder it so frequently accompanies. It has been aligned, for instance, to the delusional syndrome that afflicts those unable to recognize neurologically-wrought deficits such as blindness and paralyses. If not itself a symptom, lack of insight is at the least portrayed as *a clinical criterion *of severe disorder. The patient's psychosis is indicated by a failure to adopt what has been seen as *an accurate acknowledgement that mental disorder is present*. In his classic statement, Aubrey Lewis defined insight as "the correct attitude to morbid change in oneself, and moreover, the realization that the illness is mental " (cited in [19] page 496.)(This is not a position limited to the medical perspective, it should be added: in twelve step programs, failure to acknowledge one's status as an alcoholic, for example, is regarded as confirmation of that status.)

Recent work on insight has emphasized that failure of insight is not an all or nothing state. Moreover, there is some variation over the particular set of beliefs comprising the "lack of insight" taken to be so central to healing. Unawareness that anything is wrong, and that what is wrong indicates illness or disorder, for example, can each be distinguished from incorrect " attributions" or explanations of the causes of the illness [20].

Because an acknowledgement of one's symptoms as illness is used as a measure of health or healing, an ostensibly medical framing has immense value for the narrator. If lack of insight is a clinical indicator of disorder, then only through acknowledging the presence of illness, can we appear to be well. And this point, with all its irony, is rarely lost on the authors of these accounts. Thus, the part played by insight, it is also frequently implied, constitutes a snare. It seems to ensure a dismissive attitude towards the meaning of symptoms that is belied by the patient's own experiences - yet it has untold pragmatic benefits.

Two implications of the clinical role of insight into illness are worth noting here. First, to the extent that it represents a strategic effort to appear well, a medical "spin" might be expected to distort and obscure much of what makes these accounts valuable as descriptions of the experiences themselves. Reading between the lines may be not only desirable, but dictated by the quest for accuracy.

And second: the use of insight into illness as a criterion of healing and health is not merely a clinician's power trip, although it is sometimes cast that way both by consumers and by outside observers of psychiatric practice. Treatment that is involuntarily imposed, it is generally agreed, is less ethically satisfactory than that which is undertaken with consent. It is thus morally incumbent on medical practitioners to seek from patients their informed consent to treatment. For all that he might consent, the patient who does not recognize his symptoms and fails to accept the language and presuppositions of medical psychiatry approaches the moral status of an involuntary patient. Just as, we saw, the patient has an incentive to describe her experience through the master narrative, the ethical burden on clinicians requires them to privilege information expressed in medical terms. This challenge has the form of a clinician's dilemma, one of many associated with psychiatric practice. How it is to be resolved may require concessions on the part of both the patient (acknowledgement that all is not, or was not, well, for example), and the practitioner (admission that the medical explanations and presuppositions most pertinent to his or her practice may not be exhaustive, perhaps).

### Recognition rights and group identity

Memoirs and first person accounts of psychiatric diagnosis and mental health care offer indications about how experiences of mental disorder might be reframed and redefined as part of efforts to acknowledge and honor recognition rights and epistemic justice. The source material, containing ways to construe such experiences, and rich with implications for a more general project of reconstructive cultural semantics, is there for the taking. It seems we need only attend. Yet lingering concerns remain, expressed in additional and more recent work by Nancy Fraser where she has noted the uncomfortable fit between recognition and identity politics [21]. These concerns involve both extra group and intra group effects, we shall now see.

The politics of recognition aims to repair the mis-recognition to which some groups have been subject. In the Hegelian tradition from which these ideas derive, individual identity is constructed as dialogical, through a process of mutual recognition. A group cannot construct its identity alone, this implies. Cultural identity cannot be an "auto generated, auto description presented to others as an *obiter dictum*," in Fraser's words (See [21] page 216.) That would engender another sort of mis-recognition, and one that encouraged separatism and group enclaves - both very real risks associated with today's consumer groups.

Our task of reconstructive cultural semantics will need to engage more than the groups most immediately affected. Of course consumers must be central participants, as the slogan ("nothing about us without us") implies. But this task of reconstruction requires consumers be joined by allies from outside the group as well. These new meanings cannot be forged through *obiter dictum*, but through negotiation. This will involve doctors and other mental health service providers, as well as the rest of the community. And it will not be easy, as we saw from the examples introduced above of contested realities surrounding patient insight, and the meanings attributed to symptoms.

It remains to be seen what compromises emerge from such negotiation. But surely they will be required of all participants. For example, acknowledgement that mental disorder represents a state that all is not well, or that has disadvantages for its sufferer - a concession by the consumers - seem likely to have to be matched, as we saw, by something like the admission that the medical explanations and presuppositions are not exhaustive, and that symptoms may be meaningful, and affect outcomes, in more than one way. Arguably, what has been recognized as a *deflationary turn *in writing about psychotic symptoms augers the direction these negotiations can be expected to take. The deflationary turn includes increasing acknowledgement that some forms of hallucination and delusion are benign; that even patients lacking full insight may understand their conditions in terms that can produce healing, and that a continuum links clinical depression to more everyday unhappiness, for example - each of which deflates the pretensions of earlier-held medical psychiatric orthodoxies (See [22].)

Dangers associated with solely intra group effects are also noted by Fraser. Group-wrought definitions risk imposing a revision no less constraining than the mis-recognizing one it aims to replace. Fraser speaks of this concern as a risk that identity will be reified. Stressing the need for an authentic, self-affirming and self-generated collective identity, as she says, "puts moral pressure on individual members to conform to a given group culture," discouraging cultural dissidence, experimentation, and cultural criticism. The overall effect is then to impose a unitary, simplified group identity that denies "the complexity of people's lives, the multiplicity of their identifications, and the cross-pulls of their various affiliations" (See [21] page 215.) Some literature and rhetoric from present day consumer movements, with its simplified models of the relation between self and symptoms and of psychiatric experience, arguably exhibit the risk Fraser identifies here. If the task of reconstructive cultural semantics is to avoid these limitations, it must acknowledge and emphasize the heterogeneity of the effects of, and of responses to, psychiatric diagnosis and care.

## Conclusion

The political category of recognition rights allows us to see the extent and scope of mental health consumer movements' demands. Not only are social and economic transformation called for, so are transformed meanings and identities. My goal in this paper has been to identify and applaud the first steps towards a reconstructive cultural semantics that can be found in today's mental illness memoirs, but also to draw attention to the magnitude of the challenge involved - for consumers, practitioners, and for the rest of us - as these new meanings and realities are negotiated..

## Competing interests

The authors declare that they have no competing interests.
